# Evaluation of digital and manual orthodontic diagnostic setups in non-extraction cases using ABO model grading system: an in-vitro study

**DOI:** 10.1186/s12903-024-03961-z

**Published:** 2024-02-09

**Authors:** Sherwet Shakr, Ibrahim Negm, Hatem Saifeldin

**Affiliations:** Orthodontic Department, Faculty of Dentistry, Ain Shams University, Cairo, Egypt

**Keywords:** Orthodontic diagnostic setup, Digital models, ABO model grading system, 3D printer, 3D scanner

## Abstract

**Background:**

To evaluate the outcome quality of manual and digital orthodontic diagnostic setups in non-extraction cases according to the American Board of Orthodontics model grading system and to calculate the laboratory time needed for orthodontic diagnostic setup construction.

**Methods:**

The sample consisted of 60 pretreatment models of non-extraction orthodontic cases with age ranges of 18–30. The study models were duplicated and scanned with 3Shape R-750 scanner. Digital and manual diagnostic setups were constructed according to their respective treatment plans. Digital diagnostic setups were 3D printed and then both manual and digital setups were assessed using the modified American Board of Orthodontics Cast Radiograph evaluation score (ABO CRE), which includes alignment, marginal ridge, buccolingual inclination, occlusal contacts, occlusal relationships, interproximal contacts, and overjet. The laboratory time needed for orthodontic setups was measured in minutes.

**Results:**

The total ABO CRE score of the digital diagnostic setup group (5.93 ± 2.74) was significantly lower than that of the manual diagnostic setup group (13.08 ± 3.25). The manual diagnostic setup had significantly larger scores in marginal ridge, overjet, overbite, buccolingual inclination, occlusal relationship, and total scores (*P* < 0.01). However, the digital diagnostic setup had a statistically larger occlusal contacts score than the manual diagnostic setup (*P* < 0.01). There was no significant difference between the alignment and the interproximal contacts scores in either group. The manual diagnostic setup needed significantly longer laboratory time (187.8 ± 14.22) than the digital setup (93.08 ± 12.65) (*P* < 0.01). Comparison between broken teeth was performed by using the chi-square test which found no significant difference between different tooth types.

**Conclusions:**

Digital diagnostic setup is a reliable tool for orthodontic diagnostic setup construction providing excellent quality setup models. Manual diagnostic setup is time consuming with a technique-sensitive laboratory procedure.

## Introduction

Accurate orthodontic treatment largely depends on precise diagnosis, involving the use of multiple diagnostic tools, such as dental models and radiographs for a thorough evaluation of dental, skeletal, and soft tissues. Dealing with complicated orthodontic and orthognathic cases further necessitates accurate documentation, treatment plan simulation, and clear communication between the dental team and the patient. One tool found to be valuable for this purpose is the orthodontic diagnostic setup [1–4]. 

The orthodontic diagnostic setup was introduced in the 1940s by Kesling as the means for both proper orthodontic diagnosis and treatment [1, 2]. Orthodontic diagnostic setups also help illustrate different treatment options, limitations, teeth movement simulations, anchorage requirements, the study of teeth position three-dimensionally, and inter-arch and intra-arch discrepancies, especially in borderline, complicated, or orthognathic surgery cases [3, 4]. 

A conventional orthodontic diagnostic setup is constructed by cutting plaster study models and manually rearranging the teeth in wax dental arches to simulate treatment plan objectives [3, 4]. Even though manual diagnostic setups provide great value for orthodontic diagnosis, treatment planning, and orthodontic appliance design, they are not routinely performed at orthodontic clinics, as they require complicated and technique-sensitive procedures. They also require large storage spaces and careful handling of the casts to avoid distortion, in addition to being difficult to transfer and share between dental team members [5]. Moreover, the process of cutting plaster models involves inhaling inorganic plaster dust that could cause multiple health hazards [6, 7]. 

Dental technological advancement has resulted in the digital transformation of modern orthodontic practices, including digital orthodontic diagnostic records, appliance design, and fabrication [8, 9]. Modern orthodontic software allowed the digital design of orthodontic diagnostic setups, which utilized many tools to control 3D tooth movement and orthodontic analyses. Orthodontic tools like; intraoral Scanners, CBCT, digital treatment planning programs, digital modeling programs, and 3D printing utilities were found necessary for modern orthodontic clinical practice and hence, should be considered for orthodontic training programs [10]. 3D digital orthodontic setups can now be merged with CBCT for more accurate orthognathic simulations with good reliability [11–13]. In addition, it was found that orthodontic digital setups had clinically acceptable accuracy in predicting treatment outcomes, especially in less complicated cases [14]. 

Digital orthodontic simulation**s** are currently fundamental for treatment plan simulations, aligner design, customized lingual orthodontic brackets, indirect bonding jigs, customized wire design, and orthodontic education [15–17]. . Computer-based orthodontic simulations should be required to enhance educational impact in orthodontic education especially in post-Covid-19 pandemic era [15]. Orthodontic simulations are essential for orthognathic treatment planning and dental team communications. Lv et al. [18] found that dental specialists considered the 3D digital simulation more intuitive, provided better professional medical team communications and aided their treatment plans decision-making process. Also, patients found that 3D treatment simulation showed obvious advantages in the aspects of intuitiveness and treatment understanding and the satisfaction. A study by Hou et al. [19] revealed that viewing digital diagnostic setups increased the practitioner’s confidence levels regarding the treatment plan choice and led to changes in the treatment plan in approximately 24% of the cases.

According to Im et al. [20], digital diagnostic setups were found to have worse quality outcomes than manual diagnostic setups in total score, overjet, and occlusal contact scores. However, they reported issues regarding virtual collision detection between 3D objects in the 3Txer program (Orapix) they used in their study. Nevertheless, no other studies have compared manual and digital orthodontic setups’ quality outcomes regarding the ABO model grading system, despite the availability of more modern software with collision detection features.

Additionally, no other study has investigated laboratory time procedures needed in manual and digital diagnostic setup construction as an outcome. Therefore, the primary objective of this study is to evaluate the outcome quality of the manual and digital orthodontic diagnostic setups in non-extraction cases according to the American Board of Orthodontics model grading system. The secondary objective is to calculate the laboratory time needed for orthodontic diagnostic setup construction.

## Materials and methods

This was a retrospective comparative in-vitro study conducted in the Orthodontic department, Faculty of Dentistry, Ain Shams University.

### Ethical approval

The ethical approval for this study was submitted to the Faculty of Dentistry, Ain Shams University Ethical Committee (FDASU-ER102307). No changes were made to the methods after the study commenced.

### Sample size calculation

The sample size was determined based on a prior study by González and Teramoto [21] which compared 3D-printed virtual setup models with manual setup models, In that study, the inter-molar width of maxillary arch was reported as (55.28 ± 2.58) in the manual setup group and (54.43 ± 2.61) in the printed digital setup group. Calculations were performed using an expected difference of 1, with the power set at 80% and the type 1 error probability (alpha) associated with this test set at 0.05%. Sample size calculation was executed using G*Power version 3.1.9.7 (G*power software: Universität Düsseldorf, Germany), resulting in a predicted sample size of 55 cases. To account for any broken casts during manipulation, a total sample size of 60 cases was chosen.

### Materials

Inclusion criteria: complete case records (which included pretreatment case evaluation, extraoral and intraoral photos, lateral cephalometric and panoramic radiographs, approved treatment plan), along with pretreatment study models in good condition. Each case was required to have full permanent dentition with fully erupted teeth, excluding third permanent molars; age range from 18 to 30 years old with cervical vertebrae maturation stage 6; exhibit a skeletal class 1 jaw relationship (according to ANB and Wits appraisal normal values); have an Angle’s class I molar relationship; and have a non-extraction treatment plan.

Exclusion criteria: Cases with a history of cleft lip and/or palate; present teeth anomalies or malformations; impacted or partially erupted teeth; anterior open or deep bite; negative anterior overjet or treatment plan involving orthognathic surgery.

### Study procedure

**Fig. 1:**
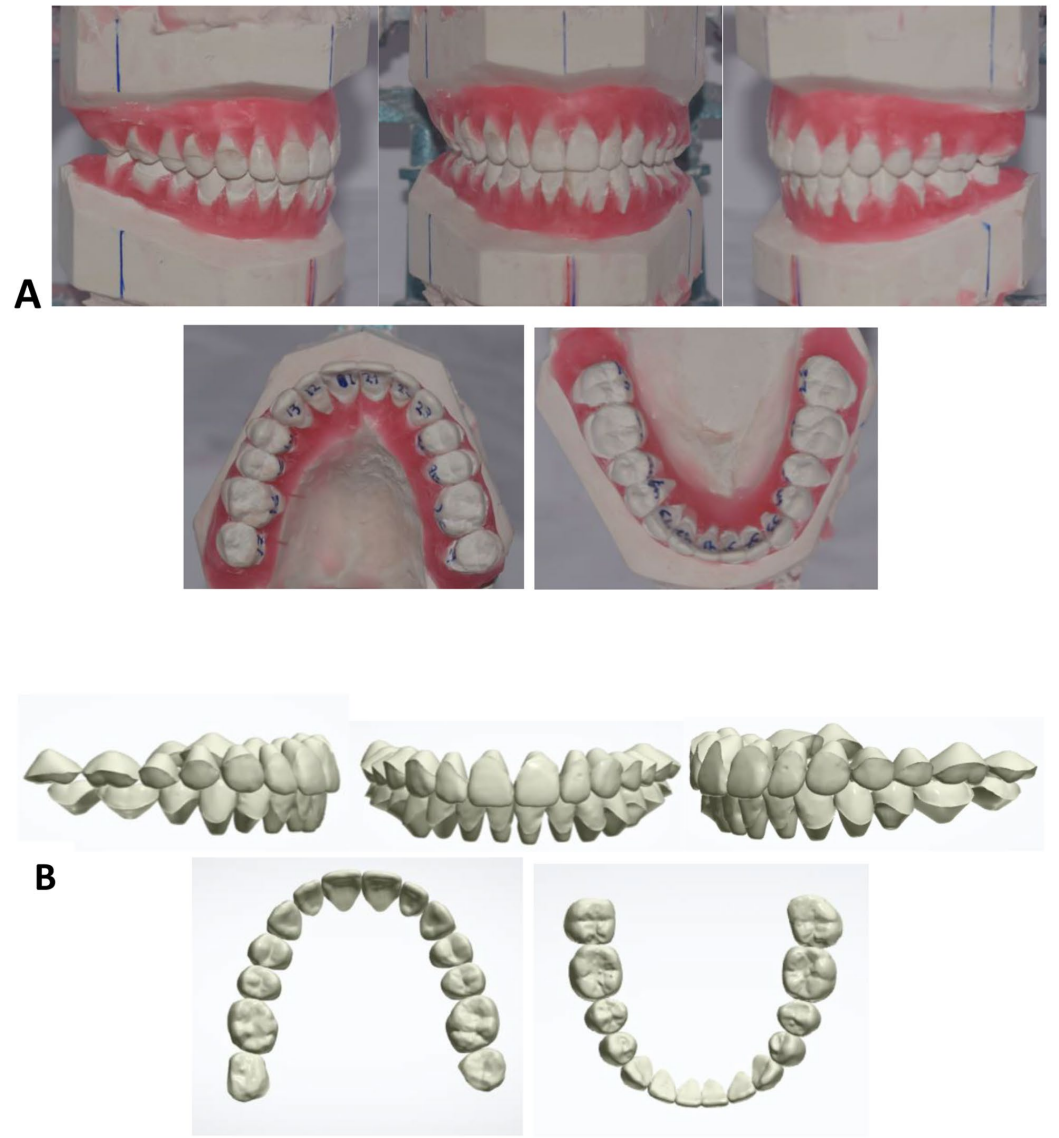
**A**: Manual orthodontic diagnostic setup. **B**: Digital orthodontic diagnostic setup

Orthodontic diagnostic setups were constructed according to their respective treatment plan, outlining anchorage requirements and incisors’ end-of-treatment goal positions. Interproximal reduction necessary to achieve the treatment plan was performed considering Bolton discrepancy and arch size discrepancy to achieve ideal positions of teeth and normal intra- and intermaxillary relations according to Andrews’ six keys of normal occlusion [22] (Fig. 1). All setups were performed by one investigator, who had proper training in both methods to ensure standardization.

### Manual diagnostic setup

The pretreatment study models were duplicated using alginate (Hydrogum 5, Zhermack, Italy) impressions of the upper and lower arch models, following the manufacturer’s instructions. The duplicates were poured after 30 min, a timeframe validated for high accuracy in studies by Rohanian et al. [23] and Aalaei et al. [24].

The duplicated pretreatment study models were scanned using a 3Shape R-750 desktop scanner (3Shape, Copenhagen, Denmark). Subsequently, they were mounted on a mean value articulator and cut to fabricate a manual orthodontic diagnostic setup, following the method demonstrated by Araújo et al. [2]. The study protocol specified that if teeth were severely damaged, the entire setup process would be repeated, starting from the duplication of the original orthodontic study models, followed by 3D scanning and preparation of the new duplicated models. Notably, no repetition was required for any of the cases during the study. The number and type of broken teeth were recorded for later analysis.

### Digital diagnostic setup

Digital diagnostic setups were constructed using the OrthoAnalyzer program (3Shape, Copenhagen, Denmark), following the protocol demonstrated by Sung et al. [8]. The digital setup was subsequently exported and saved as STL files for later review and 3D printing.

The same customized elliptical mandibular arch form, suggested by González and Teramoto [21], was employed for both manual and digital setup construction. The lower arch form was designed on OrthoAnalyzer software (3Shape, Copenhagen, Denmark) and then traced at a 1:1 scale onto acetate paper to create a transparent template for manual diagnostic setup construction, ensuring the standardization of procedures.

Digital setup STL files were imported into the Appliance Designer Program (3Shape, Copenhagen, Denmark) for trimming and the addition of ID tags to the models. The Vlare slicer program (Vlare Technology Co., Shenzhen, China) was utilized to prepare virtual diagnostic setup files for 3D printing. The STL files of the digital setups were exported to an M10-8 K LCD 3D printer (IN3D.Co, Egypt). Proshape 405 nm UV resin (Proshape Digital Solutions, Turkey), a ceramic-based water-washable LCD printer resin, was used for printing the models. The 3D models were hollowed with a 2 mm wall thickness.

### Study measurements

Manual diagnostic setup and printed digital diagnostic setup models were assessed by the modified American Board of Orthodontics Cast Radiograph Evaluation (ABO CRE) using ABO gauge. Seven out of eight criteria of the ABO CRE [25] were assessed: alignment, marginal ridge, buccolingual inclination, occlusal relationship, occlusal contact, overjet, and interproximal contact. Root parallelism, which needed a panoramic radiograph for the evaluation, was excluded. The modified ABO CRE assessment comprised various scores. The alignment score, which evaluates anterior and posterior teeth proper alignment, serves as the orthodontic setup main objective, affecting both teeth function and esthetics. The marginal ridge score evaluates the proper vertical level of the posterior teeth, cementoenamel junctions and interdental bone. The buccolingual inclination score evaluates the buccolingual inclination of the posterior teeth, which is essential to establish good occlusion, avoid balancing interferences and establish proper function. Occlusal contacts score evaluates the adequacy of posterior occlusion. The occlusal relationship score evaluates the anteroposterior relation of upper and lower posterior teeth using Angle’s relationship criteria. The overjet score evaluates the transverse relationship of the posterior teeth and the anteroposterior relationship of the anterior teeth, while interproximal contacts score checks whether the interproximal spaces between the teeth are closed.

The laboratory time needed to construct diagnostic setups was recorded using a stopwatch to the nearest minute. For the manual diagnostic setup construction, timing commenced from the initiation of diagnostic setup steps on trimmed orthodontic models and concluded upon finishing the manual diagnostic setup. This excluded the time needed for dental plaster used to mount models on an articulator to air dry.

For the digital diagnostic model construction, timing commenced after importing scanned model files to the OrthoAnalyzer program (3Shape, Copenhagen, Denmark), and then the recording process continued through the teeth segmentation process until finishing the virtual diagnostic setup.

### Statistical analysis

Statistical analysis was performed by SPSS software (version 20; IBM, Chicago, USA) and Graph Pad Prism (Graph Pad Technologies, USA.). Intra- and inter-observer reliability were investigated by repeating the modified ABO CRE score assessment of 20 randomly selected cases from the study sample. Intra-observer reliability was assessed by registering the measurements by the same investigator, two weeks after the first measurement. Inter-observer reliability was assessed by another trained and qualified investigator on the same 20 randomly selected cases. The intraclass correlation coefficient (ICC) with 95% confidence interval (CI) was used to assess the reliability, where excellent agreement (α is greater than 0.9) and very good agreement (α ranges from 0.9 to 0.8) were observed.

Shapiro Wilk and Kolmogorov-Smirnov tests were used for normality exploration, which showed that time measurements had a normal distribution (parametric data) resembling a normal Bell curve in both groups; thus, a paired t-test was used to compare laboratory time in both groups. The Wilcoxon signed rank test was used to compare the modified ABO CRE score between manual and digital setups, as data were non-parametric in both groups (*P* < 0.05). The Wilcoxon signed rank test was used to compare the modified ABO CRE score between manual and digital setups, as score data were non-parametric in both groups (*P* < 0.05). Comparison of the percentage and frequency of broken teeth was described using the chi-square test, which is used for descriptive qualitative data.

## Results

Comparison of modified ABO CRE score categories and total Score between the two groups using the Wilcoxon signed rank test is shown in (Table 1). This study revealed that the total ABO CRE score in the digital diagnostic setup group (5.93 ± 2.74) was significantly lower than that in the manual diagnostic setup group (13.08 ± 3.25).

Laboratory time was compared between the manual setup group and the digital setup group using a paired t-test, as shown in (Table 2). The findings indicate that the manual setup group (187.8 ± 14.22) had a significantly longer laboratory time than the digital setup group (93.08 ± 12.65).

Comparison between broken teeth during the construction of the manual diagnostic setup was performed by using the chi-square test (Table 3). No significant differences were found between different tooth types. The highest percentages of broken teeth, at 2.5%, were observed in the upper lateral incisors and lower central incisors, each with a frequency of 3 broken teeth per tooth type. The total percentage of broken teeth in both the upper and lower arches was 0.71%, with a total of 12 teeth broken in both arches.

Interclass coefficient (ICC) measurements, ranging from 1.00 to 0.85, indicated very good to excellent inter- and intra-observer reliabilities across all results.

**Table 1:** Comparison of modified ABO CRE score categories and total Score between the two groups. (Min.) Minimum, (Max.) maximum, (M) mean, (SD) standard deviation, (MD) mean difference

Modified ABO-CRE score	Group	Min.	Max.	M	SD	Wilcoxon signed rank test		
						MD	SD	*P* value
Alignment	**Manual**	0.00	1.00	0.12	0.32	0.10	0.35	0.07 ns
	**Digital**	0.00	1.00	0.02	0.13			
Buccolingual inclination	**Manual**	1.00	10.00	5.63	2.15	2.52	2.78	< 0.0001*
	**Digital**	0.00	8.00	3.12	1.89			
Overjet	**Manual**	0.00	5.00	1.42	1.43	1.03	1.56	< 0.0001*
	**Digital**	0.00	2.00	0.38	0.64			
Occlusal contact	**Manual**	0.00	4.00	0.37	0.80	-0.35	1.25	0.02*
	**Digital**	0.00	3.00	0.72	0.83			
Occlusal relationship	**Manual**	0.00	3.00	0.53	0.89	0.47	0.85	< 0.0001*
	**Digital**	0.00	1.00	0.07	0.25			
Marginal ridge	**Manual**	1.00	9.00	5.02	1.80	3.38	1.92	< 0.0001*
	**Digital**	0.00	7.00	1.63	1.44			
Interproximal contact	**Manual**	0.00	0.00	0.00	0.00	0.00	0.00	-------
	**Digital**	0.00	0.00	0.00	0.00			
Total ABO-Cre score	**Manual**	6.00	20.00	13.08	3.25	7.15	3.98	< 0.0001*
	**Digital**	0.00	15.00	5.93	2.74			

*; significant (*P* ≤ 0.05) ns ; non-significant (*P* > 0.05)

**Table 2:** Comparison of the Laboratory time between the two groups. (Min.) Minimum, (Max.) maximum, (M) mean, (SD) standard deviation, (MD) mean difference, (SEM) Standard error of mean and (CI) confidence interval

Measurement	Group	Min.	Max.	M	SD	Difference					
						MD	SD	SEM	95% CI		*P* value
									Lower	Upper	
Time (minute)	**Manual**	153.00	211.00	187.80	14.22	94.72	15.99	2.05	90.59	98.83	< 0.0001*
	**Digital**	66.00	121.00	93.08	12.65						

*; significant (*P* ≤ 0.05) ns ; non- significant (*P* > 0.05)

**Table 3:** Frequency and percentage of broken teeth in both upper and lower manual diagnostic setup models

Tooth type		Total number	Frequency	Percentage %	*P* value
Upper	Central incisor	120	2	1.67%	0.08 ns
	Lateral incisor	120	3	2.5%	
	Canine	120	0	0.0%	
	First premolar	120	0	0.0%	
	Second premolar	120	0	0.0%	
	First molar	120	0	0.0%	
	Second molar	120	1	0.83%	
Lower	Central incisor	120	3	2.5%	
	Lateral incisor	120	2	1.67%	
	Canine	120	0	0.0%	
	First premolar	120	0	0.0%	
	Second premolar	120	0	0.0%	
	First molar	120	0	0.0%	
	Second molar	120	1	0.83%	
Total		1680	12	0.71%	

ns: non-significant difference as *P* > 0.05

## Discussion

Orthodontic diagnostic setups play a crucial role in illustrating various aspects of treatment, including treatment options, limitations, teeth movement simulations, anchorage requirements, three-dimensional analysis of teeth positions, and assessing inter-arch and intra-arch discrepancies, particularly in complex cases like borderline and orthognathic surgery cases. Furthermore, orthodontic simulations were found critical in orthodontic education and orthognathic simulations [10–12]. Orthodontic virtual simulations were found to be clinically accurate in predicting treatment outcomes, particularly in simple orthodontic cases [14].

In this study, a sample of 60 cases was meticulously selected based on a strict inclusion and exclusion criteria to ensure a homogenous group with straightforward orthodontic treatment plans. All selected cases comprised adult patients with cervical vertebrae maturation stage 6 to eliminate the influence of remaining growth and the need for compensating for dentoalveolar and skeletal growth increments during the construction of diagnostic setups.

Both manual and digital diagnostic setup protocols were initiated by positioning the lower incisors in their end-of-treatment desired locations, guided by mandibular biological limits, lateral cephalometric radiograph findings, and the treatment plan. The alignment of the lower arch was performed while preserving the pre-treatment lower arch form, a strategy recommended by Kesling [3], Little [26], and Saifeldin et al. [27] to ensure stable orthodontic treatment outcomes.

For the digital diagnostic setup, the 3Shape R-750 desktop scanner (3Shape, Copenhagen, Denmark) was chosen to scan the duplicated models, based on previous studies by Saleh et al. [28], Lemos et al. [29], and Bukhari et al. [30]. These studies highlighted the reproducibility, accuracy, and reliability of digitally scanned models using 3Shape desktop scanners compared to traditional plaster models.

To assess the quality of orthodontic treatment, a modified ABO CRE score was employed. Digital orthodontic diagnostic setup models were 3D printed, facilitating ABO CRE evaluation with an ABO measuring gauge. This approach was chosen based on previous research by Nguyen [31] and Okunami et al. [32] which demonstrated significant variability and inaccuracies in digital measurements of ABO-OGS.

The results of this study revealed significantly lower modified ABO CRE scores and overjet scores in the digital diagnostic setup group (5.93 ± 2.74) compared to the manual diagnostic setup group (13.08 ± 3.25). Neither group achieved a total ABO CRE score exceeding 20 points, while the ABO model grading system sets the threshold score at 27. Our findings are in contrast with those of Im et al. [20], who reported significantly higher total ABO OGS scores in the digital setup group. Moreover, Im et al. [20] recorded the highest score deduction in either group as 25 points. The improved quality of diagnostic setups in this study can be attributed to the precise standardized protocol for building virtual setups, as well as the use of different software from the one employed by Im et al. [20].

Regarding marginal ridges, buccolingual inclination, and occlusal relationship scores, the digital diagnostic setup showed significantly lower scores compared to the manual diagnostic setup. These results contrasts with the findings of Im et al. [20], who reported no significant difference in these parameters. The larger ABO CRE scores in the manual setup group suggest that these categories exhibited inferior setup quality. This is attributed to the difficulty in achieving fine and precise movements of plaster teeth in viscous wax. The digital approach, on the other hand, offers superior control over tooth movement in all dimensions, allowing adjustments down to 0.01 mm and 0.01 degrees. Furthermore, modern orthodontic digital software incorporates advanced tools such as 2D cross-section analysis, 3D measurements, and collision detection, which contribute to superior digital diagnostic setup outcomes.

The present study further revealed a statistically higher occlusal contact score in the digital setup group compared to the manual diagnostic setup group. This difference is likely attributed to the inherent challenge of evaluating occlusal contacts in digital software, which lacks the tactile feedback present in manual setup procedures. However, it’s noteworthy that the interproximal contact score and the alignment score exhibited no significant variance between the manual and digital diagnostic setup groups. This consistency in results aligns with the findings reported by Im et al. [20].

While numerous studies have highlighted the time-consuming nature of manual setup compared to digital setup, to our knowledge, no other research assessed it as an outcome. Assessing the time required for orthodontic diagnostic setups is a complex task influenced by various factors, such as the learning curves of both techniques, digital literacy, operator experience and case difficulty variations. To overcome these obstacles, our study had a large sample size (*n* = 60) with strict inclusion and exclusion criteria. Our results revealed that the shortest time recorded for the digital setup was 66 min, and the longest was 121 min. However, the shortest time recorded for the manual setup was 153 min. Notably, even the most time-consuming digital setup was completed 32 min faster than the fastest manual setup. It is crucial to acknowledge that both procedures are time consuming. Moreover, it’s important to note that the manual setup laboratory time specified in our study excludes the additional time required for duplicating, trimming the models, and the setting of plaster used to mount the models on an articulator. These supplementary tasks can extend the overall time commitment significantly, requiring several hours.

These findings are consistent with other studies [4, 20, 33, 34] that highlighted the prolonged duration associated with manual setup. Barreto et al. [33] stated that the digital setup on OrthoAnalyzer (3Shape, Copenhagen, Denmark) software took approximately 2 h, while manual setup construction needed a much longer time. Araújo et al. [4], Im et al. [20] and Braga et al. [34] all shared the same finding that the digital diagnostic setup needed less laboratory time than the manual setup; however, they did not provide an estimation of how long they needed to construct the setups.

The study assessed the percentage and frequency of broken teeth during the construction of manual diagnostic setups. Among the 60 manual diagnostic setups handled, a total of 12 broken teeth were recorded. The highest frequency of broken teeth occurred in upper lateral incisors and lower central incisors, with a frequency of 3 each. This observation is likely attributed to their narrow anatomy within their respective arches. Furthermore, these teeth are commonly crowded or blocked out, posing challenges in their separation. The subsequent most frequently broken teeth were the lower lateral incisors and upper central incisors, which share characteristics of being bucco-lingually thin and more likely to be crowded, particularly in comparison to larger posterior teeth.

Manual diagnostic setup involves a technique-sensitive process that entails cutting and separating teeth from plaster models, shaping them, and arranging them in modeling wax. The inherent brittleness of plaster can make it susceptible to fracture. This may result from the wedging effect of the dental saw on the brittle model material or the presence of trapped air bubbles in the plaster model. It is noteworthy that the non-significance of the frequency of broken tooth incidents during the manual setup in our study may be attributed to the utilization of extra hard plaster, coupled with the use of a vibrator during the pouring process to expel as many air bubbles as possible. Additionally, the careful technique employed in separating and handling the teeth likely contributed to mitigating the risk of breakage.

Our study showed that digital orthodontic diagnostic setups is as reliable as manual diagnostic setup. The significantly less time needed for performing digital diagnostic setup can allow the integration of routine diagnostic setup in our diagnosis process.

### Limitation of our study

Our study focused on assessing diagnostic setup outcomes specifically in Class I malocclusion cases. While it is crucial to extend these measurements to more complex cases for a comprehensive evaluation, our initial approach aimed to standardize the investigation and minimize variables that could impact our results.

## Conclusions

1. Digital diagnostic setup is a reliable tool for orthodontic diagnostic setup construction providing excellent quality setup models.
2. Digital orthodontic diagnostic setup exhibited a lower ABO-CRE score than the manual diagnostic setup, as per the criteria of the modified ABO model grading system.
3. Manual orthodontic diagnostic setup is a time-consuming and technique-sensitive laboratory procedure.

## Data Availability

The datasets used and/or analyzed during the current study are available from the corresponding author on reasonable request.
